# Relative Importance of Race, Sex, and Non-demographic Characteristics in Orthopaedic Surgeon Selection: A Survey of U.S. Adults

**DOI:** 10.7759/cureus.103356

**Published:** 2026-02-10

**Authors:** James E Feng, Rasheed Abdullah, Muzamil Ahmad, Betina B Hinckel, Leonardo M Cavinatto

**Affiliations:** 1 Orthopaedics, Golden State Orthopaedic Surgery and Spine, Walnut Creek, USA; 2 Radiology, Corewell Health William Beaumont University Hospital, Royal Oak, USA; 3 Surgery, Oakland University William Beaumont School of Medicine, Rochester, USA; 4 Orthopedic Surgery, Corewell Health William Beaumont University Hospital, Royal Oak, USA

**Keywords:** decision making, orthopedic surgeons, patient preference, surveys and questionnaires, united states

## Abstract

Introduction: Orthopaedic surgery remains among the least diverse specialties in the United States (U.S.), and our understanding of how patients weigh surgeon demographics relative to other important selection factors remains unclear. This study sought to characterize public attitudes regarding various demographic and non-demographic attributes of orthopaedic surgeons.

Methods: A prospective, cross-sectional survey was administered to U.S. adults recruited via Amazon Mechanical Turk (age ≥18 years; Human Intelligence Task (HIT) rating >95%) and delivered through Qualtrics. Respondents rated surgeon attributes and referral sources using Likert scales. Analyses used t-tests and χ² tests (p < 0.05).

Results: A total of 510 responses were included for analysis. Support for initiatives to increase diversity was 80.8%. Respondents preferred surgeons with at least 11.2 ± 6.1 years of experience, and willingness to travel was 94.2 ± 247.0 minutes. Most respondents reported no preference for the sex (320, 62.7%) or race (352, 69.0%) of their surgeon. Among respondents who preferred a specific sex of their surgeon, men preferred male surgeons (102, 94.4%), whereas women preferred female surgeons (59, 72.0%) (p < 0.001). Regarding importance, technical competencies dominated: surgical skills were rated “most important” by 82.0% (418), and years in practice by 50.4% (257). Surgeon gender and race/ethnicity were reported as “does not matter” by 60.2% (307) and 65.1% (332), respectively. Referral from a doctor or physical therapist was rated “most important” by 72.2% (368) of respondents. Additionally, 74.3% (379) reported using the internet to find surgeons, and 78.6% (401) reported using it to research credentials.

Conclusion: Demographic concordance mattered only to a minority of respondents. Perceived factors of competence, years of experience, and trusted referral pathways outweighed surgeon sex or race in orthopaedic surgeon selection in this sample of U.S. adults.

## Introduction

Orthopaedic surgery is one of the least diverse fields of medicine in the United States, with women and racial minority groups making up a smaller proportion of the active workforce compared to other specialties [1-3]. Recent data from the American Academy of Orthopaedic Surgeons (AAOS) show that the vast majority of practicing orthopaedic surgeons are male and White, with only marginal increases in women and racial minorities over time [4]. These observations have contributed to a growing discussion surrounding the demographic makeup of orthopaedics, including potential implications for the profession and patient care, prompting initiatives to increase diversity within the field, largely through adoption of new practices in the residency selection process and increased early medical student exposure [2,5,6].

In this context, patient preferences are often centered at the forefront of discussion but are less well characterized. Patient preferences for surgeons of specific demographics, such as sex or race, may alter referral patterns, practice composition, and outward perceptions of the field. However, it remains unclear to what extent patients consider these characteristics when selecting an orthopaedic surgeon, or how frequently patients prefer surgeons who are of their own sex or race. While some studies have demonstrated that most patients do not express a strong preference for orthopaedic surgeon gender, others show that a subset of patients do, such as women who are more likely to report same-gender preferences [7-10].

Additionally, patients consider a wide range of factors when choosing a surgeon, including technical skill, communication, experience, access, and reputation, and existing studies have shown that patients rate surgeon credentials and experience as highly important [11-13]. However, when considering overall patient preferences for surgeon attributes, the existing literature is limited in defining the relative weight patients place on demographic versus non-demographic characteristics and remains poorly defined in orthopaedic surgery. Furthermore, patients increasingly use multiple sources of information to identify and evaluate surgeons, such as referrals from other physicians, recommendations from family or friends, and internet research, yet the relative importance of these care-seeking behaviors compared with demographic factors remains uncharacterized [11,14,15].

With this in mind, understanding how patients prioritize surgeon sex and race alongside other characteristics may aid ongoing discussions about the current orthopaedic workforce, particularly as new residency-selection practices continue to be adopted and evolve. Therefore, the current study sought to define patient-reported preferences for the sex and race of their orthopaedic surgeon and to assess the importance patients assign to these traits in the presence of non-demographic attributes such as surgeon training, experience, and care-seeking behaviors. We hypothesize that most respondents will rank non-demographic attributes as more important than demographic traits when selecting an orthopaedic surgeon.

## Materials and methods

In this prospective, cross-sectional survey study, a 56-question survey was developed de novo to evaluate public perceptions of the current demographics of orthopaedic surgery and how the public weighs the importance of demographics versus non-demographic characteristics of orthopaedic surgeons.

A 56-question instrument was developed and used for this purpose, combining multiple-choice questions and numeric free-text responses (see the Appendices). Initial questions collected non-identifiable respondent demographics and indirect markers of health status. Demographics included age, sex, race, primary language, education level, annual household income, U.S. region of residence (Midwest, Northeast, South, West), and regional population density (rural, low-density suburb, suburban metro, small city, large city). Health status markers included the number of hospitals within a one-hour commute, the number of primary care physician visits per year, the number of prescription medications taken per day, and a history of prior orthopaedic surgery.

Subsequent questions collected respondents’ attitudes toward diversity initiatives in orthopaedics and assessed respondents' attitudes toward various surgeon attributes, including both demographic and non-demographic characteristics, as well as care-seeking behaviors.

Study population and recruitment

Participants were recruited through Amazon Mechanical Turk (MTurk; Amazon, Seattle, Washington), a clinically validated online crowdsourcing marketplace in which “requestors” financially compensate “workers” for completing Human Intelligence Tasks (HITs), which commonly include survey-based studies [10]. Amazon MTurk consists of 250,810 active workers globally, of whom 226,500 reside in the United States [11]. To enhance data quality, recruitment was restricted to workers with a prespecified HIT rating to ensure participation by only high-quality respondents. Embedded Qualtrics security measures were enabled to prevent duplicate submissions. Inclusion criteria were age ≥18 years, residence in the United States, and an MTurk HIT rating of >95%. These criteria were enforced using MTurk worker qualifications.

All survey questions were administered using an online survey platform (Qualtrics XM, Provo, Utah). The survey required approximately 3-10 minutes to complete, and respondents received $0.50 in compensation upon completion. At the end of each Qualtrics survey, each participant was provided with a unique completion code, which they were required to enter into the MTurk interface. Completion codes were matched between Qualtrics and MTurk to ensure that each response corresponded to a unique, valid participant. Incomplete responses and entries with invalid or missing completion codes were excluded from analysis and did not receive compensation.

Statistical analysis

All analyses were performed using Anaconda (version 5.3.0; Anaconda Inc., Austin, Texas), a distribution of Python (version 3.6.6; Python Software Foundation, https://www.python.org), within the PyCharm Professional 2020.2 integrated development environment (JetBrains, Prague, Czech Republic). The primary libraries used were pandas, numpy, and their dependencies.

Primary analyses were defined a priori as comparisons of perceived importance among the sample. Continuous variables were compared between groups using Student’s two-sample t-tests. Categorical variables, including Likert distributions, were compared using chi-square (χ²) tests. A p-value <0.05 was considered statistically significant.

Ethical statement

The study protocol was reviewed by the institutional review board (IRB) and was granted exempt research status. Participation was voluntary, and all data were collected anonymously. No personal identifiers or identifying health information were collected.

## Results

Respondent demographics

In total, 510 responses were included for analysis (Table 1). The mean respondent age was 40.4 ± 11.8 years. Males comprised 54.1% (276) of respondents, females 45.3% (231), and 0.6% (3) declined to report their sex. White respondents made up 83.1% (424) of the total sample, with 7.8% (40) being Black, 7.1% (36) Asian, 0.4% (2) Native American, and 0.2% (1) Native Hawaiian or Pacific Islander. Ethnically, 92.5% (472) of the sample were not Hispanic or Latino. English was the primary language for 98.6% (503) of the sample, and 62.9% (321) reported holding a bachelor’s degree or higher. Regarding income, 72.9% (372) reported earning between $25,000 and $99,000 per year. The distribution of respondents across the United States was fairly even (24.5% (125) Midwest, 23.7% (121) Northeast, 40.8% (208) South, 10.6% (54) West), and respondents were evenly spread across urban, suburban, and rural population settings. The majority of respondents held private insurance (309, 60.6%), with the remainder covered by Medicaid (56, 11.0%), Medicare (55, 10.8%), marketplace plans (35, 6.9%), or uninsured (41, 8.0%). On average, respondents reported 6.7 ± 6.6 hospitals within a one-hour commute, 2.9 ± 5.0 physician visits per year, 1.3 ± 2.8 prescription medications per day, and 32.5% indicated a history of prior orthopaedic surgery.

**Table 1:** Baseline characteristics of survey respondents Demographic characteristics of survey respondents (N=510). Continuous variables are presented as mean ± standard deviation (SD); categorical variables are presented as counts (N) and percentages (%).

Demographic	Respondents (N=510)
Total	510 (100.0%)
Age, years	40.4±11.8
Sex	
Female	231 (45.3%)
Male	276 (54.1%)
Prefer not to say	3 (0.6%)
Race	
American Indian or Alaskan Native	2 (0.4%)
Asian	36 (7.1%)
Black or African American	40 (7.8%)
Native Hawaiian or Pacific Islander	1 (0.2%)
White or Caucasian	424 (83.1%)
Other	7 (1.4%)
Ethnicity	
Hispanic or Latino	38 (7.5%)
Not Hispanic or Latino	472 (92.5%)
Primary language	
English	503 (98.6%)
Spanish	4 (0.8%)
Other	3 (0.6%)
Education	
High school, GED, or equivalent	49 (9.6%)
Some college	81 (15.9%)
Associate’s/vocational degree	55 (10.8%)
Bachelor’s degree	232 (45.5%)
Master’s degree	71 (13.9%)
Doctorate’s degree	18 (3.5%)
Annual income	
$0-24,999	71 (13.9%)
$25,000-49,999	141 (27.6%)
$50,000-74,999	145 (28.4%)
$75,000-99,999	86 (16.9%)
$100,000-149,000	44 (8.6%)
$150,000-199,999	17 (3.3%)
$200,000+	6 (1.2%)
Region	
Midwest	125 (24.5%)
Northeast	121 (23.7%)
South	208 (40.8%)
West	54 (10.6%)
Regional population density	
Large city	91 (17.8%)
Small city	95 (18.6%)
High-density suburb	120 (23.5%)
Low-density suburb	120 (23.5%)
Rural	84 (16.5%)
Insurance status	
Private	309 (60.6%)
Medicare	55 (10.8%)
Medicaid	56 (11.0%)
Marketplace (ACA)	35 (6.9%)
Tricare	8 (1.6%)
Other	6 (1.2%)
Uninsured	41 (8.0%)
Health status	
Number of hospitals within 1 hour commute	6.7±6.6
Number of doctor visits per year	2.9±5.0
Prescription medications/day	1.3±2.8
History of prior orthopaedic surgery	166 (32.5%)

Preferences for surgeon demographics and experience

On average, respondents preferred orthopaedic surgeons with 11.2 ± 6.1 years of experience and were willing to travel 94.2 ± 247.0 minutes to see one, with substantial variation in travel time (Table 2). Regarding the current demographic makeup in orthopaedics, 80.8% (412) of respondents expressed support for initiatives to increase diversity within the field. Regarding preferred surgeon age, 57.1% (291) preferred a surgeon aged 41-50 years, 32.0% (163) preferred 51-60 years, 9.6% (49) preferred 30-40 years, and 1.4% (7) preferred 61-70 years.

**Table 2:** Respondent preferences in orthopaedic surgeons Overall respondent preferences for orthopaedic surgeons. Continuous variables are presented as mean ± standard deviation (SD); categorical variables are presented as counts (N) and percentages (%). When present, p-values derived from chi-square tests.

Item	Respondents (N=510)	p
Work experience of surgeon, years	11.2±6.1	
Time willing to travel to see surgeon (min)	94.2±247.0	
Support for increased diversity initiatives in orthopaedics	412 (80.8%)	
Preferred surgeon age		
30–40 years old	49 (9.6%)	
41–50 years old	291 (57.1%)	
51–60 years old	163 (32.0%)	
61–70 years old	7 (1.4%)	
Surgeon sex preference		
Female surgeon	65 (12.7%)	
Male surgeon	125 (24.5%)	
No preference	320 (62.7%)	
Surgeon sex preference by respondent sex		<0.001
Male respondent preference for male surgeon	102 (20.0%)	
Male respondent preference for female surgeon	6 (1.2%)	
Female respondent preference for male surgeon	23 (4.5%)	
Female respondent preference for female surgeon	59 (11.6%)	
Surgeon race preference		
American Indian or Alaskan Native	0 (0.0%)	
Asian	19 (3.7%)	
Black or African American	16 (3.1%)	
Native Hawaiian or Pacific Islander	1 (0.2%)	
White or Caucasian	122 (23.9%)	
No preference	352 (69.0%)	
Same race preference		0.390
Preference for same race, overall	141 (27.6%)	
White respondent preferring White surgeon	116/424 (27.4%)	
Black respondent preferring Black surgeon	15/40 (37.5%)	
Asian respondent preferring Asian surgeon	10/36 (27.8%)	

Regarding the preferred sex of their orthopaedic surgeon, 62.7% (320) reported no sex preference, while 24.5% (125) preferred a male surgeon and 12.7% (65) preferred a female surgeon. Of the 190 respondents who expressed a sex preference, male respondents (n = 108) overwhelmingly preferred male surgeons (102, 94.4%), and female respondents (n = 82) largely preferred female surgeons (59, 72.0%), with these preferences differing significantly (p < 0.001). Regarding preferred surgeon race, 69.0% (352) reported no specific racial preference, while 23.9% (122) preferred a White surgeon, 3.7% (19) preferred an Asian surgeon, and 3.1% (16) preferred a Black surgeon. Same-race preferences were reported by 27.6% (141) of the sample and were highest among Black (15, 37.5%), Asian (10, 27.8%), and White (116, 27.4%) respondents (p = 0.390).

Perceived importance of surgeon characteristics

When presented with specific surgeon attributes and asked to rate the importance of each, respondents largely prioritized professional experience and skill over demographic characteristics (Table 3). Surgical skills were the most highly rated attribute, with 82.0% (418) of respondents rating it as “most important” and an additional 13.5% (69) as “very important.” Years in practice was also frequently rated as of high importance, with 50.4% (257) selecting “most important” and 39.2% (200) “very important.” Similarly, bedside manner was rated “most important” by 29.8% (152) and “very important” by 35.9% (183) of respondents.

**Table 3:** Perceived importance of surgeon characteristics from the patient perspective Ratings of the importance of various surgeon attributes on a five-point Likert scale. Data presented as counts (N) and percentages (%).

Item	Most important (N=510)	Very important (N=510)	Somewhat important (N=510)	Low importance (N=510)	Does not matter (N=510)
College attended	95 (18.6%)	157 (30.8%)	146 (28.6%)	80 (15.7%)	32 (6.3%)
Medical school attended	182 (35.7%)	160 (31.4%)	110 (21.6%)	45 (8.8%)	13 (2.5%)
Fellowship attended	94 (18.4%)	166 (32.5%)	139 (27.3%)	72 (14.1%)	39 (7.6%)
Number of scientific publications	31 (6.1%)	65 (12.7%)	136 (26.7%)	197 (38.6%)	81 (15.9%)
Recognition/awards	36 (7.1%)	99 (19.4%)	200 (39.2%)	123 (24.1%)	52 (10.2%)
Affiliated hospital academic ranking	80 (15.7%)	188 (36.9%)	164 (32.2%)	55 (10.8%)	23 (4.5%)
Affiliation with college or professional team	42 (8.2%)	93 (18.2%)	138 (27.1%)	105 (20.6%)	132 (25.9%)
Years in practice	257 (50.4%)	200 (39.2%)	41 (8.0%)	9 (1.8%)	3 (0.6%)
Bedside manners	152 (29.8%)	183 (35.9%)	112 (22.0%)	43 (8.4%)	20 (3.9%)
Surgical skills	418 (82.0%)	69 (13.5%)	18 (3.5%)	2 (0.4%)	3 (0.6%)
Age	29 (5.7%)	97 (19.0%)	201 (39.4%)	125 (24.5%)	58 (11.4%)
Gender	11 (2.2%)	32 (6.3%)	47 (9.2%)	113 (22.2%)	307 (60.2%)
Race/ethnicity	15 (2.9%)	17 (3.3%)	42 (8.2%)	104 (20.4%)	332 (65.1%)
Religious background	9 (1.8%)	26 (5.1%)	28 (5.5%)	97 (19.0%)	350 (68.6%)
Community service	14 (2.7%)	31 (6.1%)	72 (14.1%)	152 (29.8%)	241 (47.3%)
Reputation among friends, family, and community	90 (17.6%)	161 (31.6%)	130 (25.5%)	64 (12.5%)	65 (12.7%)
Distance from your home	54 (10.6%)	146 (28.6%)	171 (33.5%)	87 (17.1%)	52 (10.2%)
Online reviews	96 (18.8%)	171 (33.5%)	163 (32.0%)	57 (11.2%)	23 (4.5%)

In comparison, demographic characteristics were generally rated as of low importance or as not mattering at all. For surgeon gender, only 2.2% (11) of respondents rated it as “most important” and 6.3% (32) rated it as “very important,” whereas 60.2% (307) indicated that gender “does not matter.” Surgeon race/ethnicity was also seen as of low importance, with 2.9% (15) rating it as “most important” and 3.3% (17) “very important,” while 65.1% (332) reported that race/ethnicity “does not matter.” Religious background was similarly of low importance, with 68.6% (350) of respondents indicating it “does not matter.” Surgeon age was more commonly rated as “somewhat important” by 39.4% (201) and of “low importance” by 24.5% (125), with 11.4% (58) indicating it “does not matter.”

Respondents also weighted contextual and reputation factors. Reputation among friends and family was rated “most” or “very” important by 49.2% (251) of respondents. Distance from home was rated “most” or “very” important by 39.2% (200) and “does not matter” by 10.2% (52). Online reviews were rated “most important” by 18.8% (96) and “very important” by 33.5% (171), with 4.5% (23) indicating “does not matter.”

Referral sources and surgeon selection

When considering how patients learn about orthopaedic surgeons, respondents placed the greatest importance on physician and familial referrals (Table 4). Referral from a healthcare provider was rated as the “most important” way to find a surgeon by 72.2% (368) of respondents and “very important” by an additional 21.8% (111). Recommendations from friends, family, or coworkers were similarly highly valued, with 23.3% (119) rating them as “most important” and 69.8% (356) as “very important.” In contrast, only a small percentage of respondents viewed social media or traditional advertising as important referral sources. Social media (e.g., Facebook, LinkedIn, YouTube) was rated as “most” or “very” important by 6.8% (35) of respondents and as of “low importance” or “does not matter” by 93.2% (475). Traditional advertisements (e.g., mail, television, radio) were rated as “most” or “very” important by 6.1% (31) and as of “low importance” or “does not matter” by 93.9% (479).

**Table 4:** Perceived importance of referral sources when selecting an orthopaedic surgeon Importance assigned to different referral sources. Responses are reported on an ordinal importance scale from “most important” to “does not matter” as counts (n) and percentages (%).

Item	Most important (N=510)	Very important (N=510)	Low importance (N=510)	Does not matter (N=510)
Recommendation from friends, family, or coworkers	119 (23.3%)	356 (69.8%)	26 (5.1%)	9 (1.8%)
Social media (Facebook, LinkedIn, YouTube, etc.)	13 (2.5%)	22 (4.3%)	290 (56.9%)	185 (36.3%)
Traditional advertisement (mail, TV, radio, etc.)	10 (2.0%)	21 (4.1%)	182 (35.7%)	297 (58.2%)
Referral from your doctor or physical therapist	368 (72.2%)	111 (21.8%)	12 (2.4%)	19 (3.7%)

Internet use and online resources

Most respondents indicated that they would use the internet in some fashion to aid in selecting an orthopaedic surgeon (Table 5). Of the respondents, 74.3% (379) indicated using the internet to find new surgeons, and 78.6% (401) reported using the internet to research a surgeon’s skills and credentials. Among specific online resources utilized, 73.5% (375) reported using Google, 33.5% (171) Healthgrades, 22.0% (112) Yelp, and 12.0% (61) ZocDoc, whereas 17.6% (90) indicated not using internet resources at all.

**Table 5:** How respondents use the internet to select and evaluate orthopaedic surgeons Self-reported use of the internet and specific online resources utilized to find new surgeons and to research surgeon skills. Values presented as counts (n) and percentages (%).

Use of internet in surgeon selection	n (%)
Uses internet to find new surgeons	379 (74.3%)
Uses internet to research surgeon credentials	401 (78.6%)
Internet resources utilized	
Google	375 (73.5%)
Healthgrades	171 (33.5%)
None	90 (17.6%)
Other	6 (1.2%)
Yelp	112 (22.0%)
ZocDoc	61 (12.0%)

## Discussion

In this sample of 510 U.S. adults, most respondents reported no preference for the sex or race of their orthopaedic surgeon, with the overwhelming number of respondents prioritizing technical skills, experience, and interpersonal qualities over demographic characteristics. The large majority of participants indicated no preference for surgeon sex or race, and only about a quarter expressed same-race preferences, highest among Black respondents. At the same time, 80.8% (412) of respondents indicated support for increasing diversity initiatives in orthopaedics. Taken together, the observed findings suggest that within this sample, patient preferences about surgeon demographics were relatively modest and were secondary to non-demographic attributes that patients perceived as more important when selecting an orthopaedic surgeon.

Our results are largely consistent with prior orthopaedic studies, which similarly report that a large majority of patients do not express strong preferences for surgeon sex or race, and among those that do, patients often prefer a surgeon of their own sex or racial background [7-10]. The data in the current study align with these patterns in demonstrating a large prevalence of "no preference" across both sex and race, while still identifying a minority of respondents who have same-race preferences. These findings extend the existing literature by evaluating both sex and race preferences within the same cohort and explicitly quantifying same-sex and same-race preferences while embedded within a wider assessment of preferred surgeon attributes and care-seeking behaviors.

The finding that surgeon skills and years in practice were rated as "most" or "very" important by nearly all respondents, whereas sex and race were most commonly rated as "low importance" or "does not matter," suggests that patients in this sample view surgeon demographics as a secondary determinant of choice. This observation is in line with prior studies, which also find that in patient samples, focused expertise, outcomes, and communication were attributes most frequently cited as important when selecting a surgeon [11,12]. Our data further show that bedside manner, reputation, familial recommendations, and online research also occupy a higher tier of perceived importance over demographic traits. These observed patterns may be relevant in ongoing conversations surrounding diversity in orthopaedics by suggesting that patients may place greater emphasis on markers of competence, experience, and communication, rather than demographic traits, when considering surgeon selection.

With regard to same-sex and same-race preferences, notable findings were present. Among respondents who expressed a sex preference, male respondents overwhelmingly preferred male surgeons and female respondents largely preferred female surgeons. A sizable subset within each racial group preferred a surgeon of the same race, but this did not vary significantly across racial groups. These observed patterns highlight heterogeneity in this sample’s views and how preferences for sex and race may vary depending on an individual’s background. These findings suggest that demographic concordance is important to a meaningful minority of patients, even if it is not the dominant attitude of the population overall.

Regarding how patients find and evaluate surgeons, respondents indicated that referrals from other physicians and recommendations from close contacts were the most important sources of information, consistent with existing literature [15]. Additionally, respondents largely indicated turning to the internet to research surgeons, primarily using search engines and ratings websites, and less so traditional media and social media. These findings suggest that the bulk of patient information regarding surgeon selection is encountered through primary care physicians, personal networks, and online research, rather than targeted advertising or social media, with respondents ranking these care-seeking behaviors as more important than demographic traits when selecting a surgeon.

The findings of the current study should be interpreted in the context of ongoing diversity initiatives and evolving residency selection practices in orthopaedic surgery. Initiatives to promote diversity have raised questions about how changes to orthopaedic residency selection processes may be perceived by the patient population. The current study does not evaluate the effects of specific initiatives, nor does it attempt to define any particular patient preferences as desirable. Rather, it describes how one sample of U.S. adults prioritizes sex and race of their surgeon relative to other characteristics. In this sample, most respondents endorsed increasing diversity in orthopaedics while simultaneously indicating that non-demographic characteristics, particularly technical competency and experience, were of greater importance when choosing an orthopaedic surgeon. This study does not intend to judge whether initiatives to increase diversity are more or less important. Instead, it aims to characterize how a sample of U.S. adults weigh surgeon demographics alongside other relevant attributes such as training, experience, and communication. How these findings are incorporated into policy decisions, residency selection practices, or institutional messaging lies outside the scope of this study, but they may be useful in providing contextual data for these discussions.

Several limitations should be acknowledged. First, the data are cross-sectional and based on respondent self-reports, capturing stated preferences rather than observed behaviors. Second, responses may be influenced by social desirability bias, particularly for questions regarding race and sex. Third, although the study sample was fairly even in regional and population setting distributions, it may not be fully representative of orthopaedic patient populations in different settings or countries. Fourth, the vast majority of the study sample had higher education, which could have impacted the findings. Fifth, some racial groups were small and underrepresented, possibly influencing the observed prevalence of racial preferences within those groups. Lastly, the survey is limited in that it cannot assess all potentially relevant factors influencing surgeon selection, and the relative importance of demographic preferences may differ depending on the clinical setting, such as trauma, pediatrics, or joint care.

## Conclusions

The findings from this sample of U.S. adults demonstrated that most respondents did not report strong preferences for orthopaedic surgeon sex or race and tended to rank professional skills, experience, and interpersonal qualities as of higher importance than sex or race when considering surgeon selection. At the same time, a smaller subset of respondents expressed same-sex or same-race preferences, which varied depending on respondent demographics. Most respondents reported physician referrals, family recommendations, and internet search engines as the most important means of identifying and evaluating surgeons. These findings may inform ongoing discussions surrounding diversity in orthopaedics by providing a comprehensive patient perspective on how surgeon demographics are considered alongside other characteristics in the context of what patients determine to be most important when selecting an orthopaedic surgeon.

## Appendices

**Table 6:** Questionnaire used in the study

Demographic Information
What is your age (years)?
What gender do you identify with?
What race do you identify with? (Select one.)
If “Other,” please specify:
Are you of Hispanic or Latino origin?
What is your highest level of education?
What is your annual income?
What is your primary language?
If “Other,” please specify:
In which U.S. state or territory do you reside? (50 states, D.C., Puerto Rico)
What best describes the area in which you reside?
How many different prescription medications do you take in a day?
On average, how many times do you visit a doctor per year?
What type of health insurance do you have?
Orthopaedic History & Preferences
Have you undergone any orthopaedic surgery in the past (e.g., arthroscopy, fracture repair, joint replacement, ACL reconstruction)?
If yes, what kind of orthopaedic surgery?
After reading the provided information on orthopaedic training: What would your ideal orthopaedic surgeon’s age be?
How many years of work experience would you like your orthopaedic surgeon to have?
Views on Workforce Diversity
Do you agree with the orthopaedic community's commitment to increasing workforce diversity?
Is it acceptable for an orthopaedic residency to accept a more diverse applicant in place of a less diverse but more competitive-appearing applicant, given the current evaluation system?
If a standardized surgical skills exam accurately assessed competency, is it justified for a residency to accept:
A more diverse applicant (Applicant 1) over a less diverse applicant (Applicant 2), when both applicants have similar surgical skills?
Is it justified to accept:
A more diverse applicant with satisfactory surgical skills (Applicant 1) over a less diverse but more highly skilled applicant (Applicant 2)?
Is it justified to accept:
A diverse applicant with below satisfactory surgical skills (Applicant 1) over a less diverse but more highly skilled applicant (Applicant 2)?
Importance of Surgeon Characteristics
Please rate the importance of each characteristic:
College attended
Medical school attended
Fellowship attended
Number of scientific publications
Recognition / Awards
Affiliated hospital academic ranking
Affiliation with college or professional sports team
Years in practice
Bedside manner
Surgical skills
Age
Gender
Race / Ethnicity
Religious background
Community service
Reputation among friends, family, and community
Distance from your home
Online reviews
Views on Training & Access
How do you view foreign-trained orthopaedic surgeons?
How close is the nearest hospital to you (in minutes)?
If you were to undergo an orthopaedic procedure, how far would you be willing to travel to go to your ideal orthopaedic surgeon (in minutes)?
How many hospitals are within a 1-hour commute from you?
Surgeon Demographic Preferences
What gender would you prefer your orthopaedic surgeon to be?
What race would you prefer your orthopaedic surgeon to be? (Select one.)
If “Other,” please specify.
Factors Influencing Surgeon Selection
Please rank the following in order of importance (most important at the top):
Recommendation from friends, family, or coworkers
Social media (Facebook, LinkedIn, YouTube, etc.)
Traditional advertisement (Mail, TV, Radio, etc.)
Referral from your doctor or physical therapist
Internet Use
Do you use the internet to find new surgeons before visiting them?
Do you use the internet to research your surgeon's résumé/background before visiting them?
Have you used any online physician review sites? (Select all that apply.)
If “Other,” please specify.

## References

[1] Caldwell LS, Glass N, Guyton GP, Elstein DW, Nelson CL (2025). An updated demographic profile of orthopaedic surgery using a new ABOS data set. JB JS Open Access.

[2] Payares M (2023). The current state of diversity in orthopaedics. J Pediatr Soc North Am.

[3] Kuhns B, Haws BE, Kaupp S, Maloney MD, Carmody EE, Mannava S (2022). Academic orthopaedics as a driver of gender diversity in the orthopaedic workforce: a review of 4,519 orthopaedic faculty members. J Am Acad Orthop Surg Glob Res Rev.

[4] (2025). A snapshot of U.S. orthopaedic surgeons: results from the 2018 OPUS survey. https://www.aaos.org/aaosnow/2019/sep/youraaos/youraaos01/.

[5] Gebhardt MC (2007). Improving diversity in orthopaedic residency programs. J Am Acad Orthop Surg.

[6] Schurhoff NA, Mosher H, Remer HB, Sacher C, Adalbert J, Hernandez GM (2025). The current state of diversity, equity, and inclusion (DEI) in orthopedics at the medical student level: a systematic review. J Surg Educ.

[7] Abghari MS, Takemoto R, Sadiq A, Karia R, Phillips D, Egol KA (2014). Patient perceptions and preferences when choosing an orthopaedic surgeon. Iowa Orthop J.

[8] Dineen HA, Patterson JM, Eskildsen SM, Gan ZS, Li Q, Patterson BC, Draeger RW (2019). Gender preferences of patients when selecting orthopaedic providers. Iowa Orthop J.

[9] Iguina-González E, Olivella G, Ramos-Vicente A (2022). Orthopedic provider gender preference among patients in an orthopedic surgery residency program of a Hispanic American community. Womens Health Rep (New Rochelle).

[10] Chen M, Strony JT, Kroneberger EA (2023). Patient preferences and perceptions of provider diversity in orthopaedic surgery. J Bone Joint Surg Am.

[11] Manning BT, Bohl DD, Saltzman BM (2017). Factors influencing patient selection of an orthopaedic sports medicine physician. Orthop J Sports Med.

[12] Engler ID, Ahrendt GM, Curley AJ, Musahl V (2022). Surgeon personality, time spent with the patient, and quality of facilities are the most important factors to patients in selecting an orthopaedic sports medicine surgeon. Arthrosc Sports Med Rehabil.

[13] Mirza K, Acharya PU, Crasta N, Austine J (2023). The ideal orthopaedic surgeon: comparing patient preferences of surgeon attributes to notions held by orthopaedic postgraduates. Indian J Orthop.

[14] Hoang V, Parekh A, Sagers K, Call T, Howard S, Hoffman J, Lee D (2022). Patient utilization of online information and its influence on orthopedic surgeon selection: cross-sectional survey of patient beliefs and behaviors. JMIR Form Res.

[15] Malige A, Matullo KS (2020). The influence of physician-rating websites on patient physician preference. Clin Orthop Surg.

